# A question of information mismatch in the SPC and PIL on the effect of ADHD stimulant medications on tourette's syndrome

**DOI:** 10.1192/bjo.2021.857

**Published:** 2021-06-18

**Authors:** Idura Hisham, Shazia Shabbir

**Affiliations:** 1 St George's Uni of London; 2 Frimley Park Hospital

## Abstract

**Aims:**

To assess the quality of information provided by pharmaceutical companies to patients and doctors regarding the impact of stimulant medications indicated for the treatment of Attention Deficit Hyperactive Disorder (ADHD) on Tourette's syndrome(TS) and tics in children and its implication on treatment.

**Background:**

It is estimated that between 35% to 90% of TS patients also have ADHD. However, there remains a pervasive belief that the use of stimulants to treat ADHD symptoms in children with comorbid tic disorders is contraindicated because of concerns about possible tic exacerbation. Recent studies has disproved this, which is reflected in United Kingdom(UK) and European ADHD and TS guidelines. Pharmaceutical companies are legally required to provide a Summary of Product Characteristic (SPC) and Patient Information Leaflet (PIL) for each medicine as it is an integral part of the marketing authorisation approval. The SPC contains vital information for the usage and prescription of a drug for use by healthcare professionals. The PIL included in the medication packaging is a patient-friendly version of the SPC.

**Method:**

The available stimulant medications licenced for use in paediatric patients with ADHD in the UK were identified through the Medicines & Healthcare products regulatory Agency (MHRA) website. The SPC and PIL were then accessed from the Electronic Medicines Compendium (EMC) website. Those not on the site were obtained directly from the marketing authorisation holder. Any direct mention of tics or Tourette's in the contraindication, warning and caution, or side effect section were documented. The information was then tabulated and compared.

**Result:**

Of the three stimulant drug types, 17 variations are currently available for use in the UK. There were inconsistencies found between the SPC and PIC in reference to the impact of these drugs on tics and TS in all 17 licensed medication. Most discrepancy was found in regard to TS as a side effect (16/17) and also tics (15/17). TS is also listed as a contraindication in the SPC and PIL for all available variety of Dexamphetamine class drugs. This is inconsistent with current clinical evidence and guidelines.

**Conclusion:**

The disparities in information regarding the impact of stimulant medications on tics and TS can have wide ranging effects. Outcomes could include poor patient adherence, or prevention of initiation of potentially beneficial treatment. It would benefit to standardize the information between these two documents to minimize inconsistencies in understanding between doctor and patient.

